# Genome-Wide Identification and Expression Analysis of the WRKY Gene Family in Cassava

**DOI:** 10.3389/fpls.2016.00025

**Published:** 2016-02-05

**Authors:** Yunxie Wei, Haitao Shi, Zhiqiang Xia, Weiwei Tie, Zehong Ding, Yan Yan, Wenquan Wang, Wei Hu, Kaimian Li

**Affiliations:** ^1^Hainan Key Laboratory for Sustainable Utilization of Tropical Bioresources, College of Agriculture, Hainan UniversityHaikou, China; ^2^Key Laboratory of Biology and Genetic Resources of Tropical Crops, Institute of Tropical Bioscience and Biotechnology, Chinese Academy of Tropical Agricultural SciencesHaikou, China

**Keywords:** abiotic stress, cassava, gene expression, RNA-seq, WRKY transcription factor

## Abstract

The WRKY family, a large family of transcription factors (TFs) found in higher plants, plays central roles in many aspects of physiological processes and adaption to environment. However, little information is available regarding the WRKY family in cassava (*Manihot esculenta*). In the present study, 85 *WRKY* genes were identified from the cassava genome and classified into three groups according to conserved WRKY domains and zinc-finger structure. Conserved motif analysis showed that all of the identified MeWRKYs had the conserved WRKY domain. Gene structure analysis suggested that the number of introns in *MeWRKY* genes varied from 1 to 5, with the majority of *MeWRKY* genes containing three exons. Expression profiles of *MeWRKY* genes in different tissues and in response to drought stress were analyzed using the RNA-seq technique. The results showed that 72 *MeWRKY* genes had differential expression in their transcript abundance and 78 *MeWRKY* genes were differentially expressed in response to drought stresses in different accessions, indicating their contribution to plant developmental processes and drought stress resistance in cassava. Finally, the expression of 9 *WRKY* genes was analyzed by qRT-PCR under osmotic, salt, ABA, H_2_O_2_, and cold treatments, indicating that *MeWRKYs* may be involved in different signaling pathways. Taken together, this systematic analysis identifies some tissue-specific and abiotic stress-responsive candidate *MeWRKY* genes for further functional assays *in planta*, and provides a solid foundation for understanding of abiotic stress responses and signal transduction mediated by WRKYs in cassava.

## Introduction

The WRKY family is a large family of transcription factors (TFs) found in higher plants (Rushton et al., 2010). *SPF1*, the first reported WRKY transcription factors, plays crucial roles in the regulation of gene expression (Ishiguro and Nakamura, 1994). WRKY TFs contain one or two WRKY domains which have a highly conserved WRKYGQK motif at the N-terminus and a zinc-finger structure at the C-terminus (Llorca et al., 2014). Based on the variation in WRKY domain and the pattern of the zinc-finger motif, WRKY proteins can be divided into three major groups (1, 2, and 3) with several subgroups (Eulgem et al., 2000). The group 1 typically contains two WRKY domains including a C_2_H_2_ motif, while group 2 and group 3 are characterized by a single WRKY domain. Group 2 also contains a C_2_H_2_ zinc-finger motif and can be further divided into five subgroups (2a–2e) based on the phylogeny of the WRKY domains, whereas group three contains a zinc-finger-like motif ending with C_2_-H-C (Eulgem et al., 2000).

There is considerable evidence showing that WRKY proteins play central roles in various aspects of physiological processes and adaption to the environment (Rushton et al., 2010; Ling et al., 2011), including senescence (Robatzek and Somssich, 2002; Han et al., 2014), trichome development (Johnson et al., 2002), embryogenesis (Lagacé and Matton, 2004), seed dormancy and germination (Xie et al., 2007), root development (Devaiah et al., 2007), and response to biotic stresses including bacterial (Oh et al., 2006; Xu et al., 2006; Zheng et al., 2007; Tao et al., 2009; Hwang et al., 2011; Choi et al., 2014), fungal (Li et al., 2006; Xu et al., 2006; Liu et al., 2014; Ye et al., 2014; Cheng et al., 2015), viral pathogens (Oh et al., 2006; Huh et al., 2015), and insects (Grunewald et al., 2008; Skibbe et al., 2008).

In recent years, accumulated evidence has confirmed that a large number of *WRKY* genes are induced by abiotic stresses and play important roles in the regulation of plant tolerance to abiotic stress (Seki et al., 2002; Rushton et al., 2010; Li et al., 2011; Scarpeci et al., 2013). In Arabidopsis, *AtWRKY30* was induced by abiotic stress including treatments with methyl viologen (MV), H_2_O_2_, arsenic, drought, NaCl, and mannitol, and overexpression of *AtWRKY30* increased plants tolerance to MV and salinity stresses (Scarpeci et al., 2013). *WRKY46*, another *WRKY* gene from Arabidopsis, was significantly induced by drought, salt, and H_2_O_2_, and *wrky46* mutant was less tolerant to osmotic and salt stress than WT (Ding et al., 2014). *WRKY25* and *WRKY26* were induced under heat stress and were confirmed to play positive roles thermotolerance in Arabidopsis (Li et al., 2011). Additionally, overexpression of *WRKY25* or *WRKY33* increased plant tolerance to salt stress and sensitivity to ABA (Jiang and Deyholos, 2009). Likewise, 41 out of 103 rice *WRKY* genes showed significant differences in their transcript abundance under abiotic stress (cold, drought and salinity; Ramamoorthy et al., 2008). Some rice *WRKYs* have been shown to be positive regulators of abiotic stresses, such as *OsWRKY5* (Berri et al., 2009), *OsWRKY7* (Ramamoorthy et al., 2008), *OsWRKY11* (Wu et al., 2009), *OsWRKY43* (Berri et al., 2009), *OsWRKY45* (Qiu and Yu, 2009), and *OsWRKY47* (Raineri et al., 2015). For example, overexpression of *OsWRKY45* in *Arabidopsis* was found to increase plant tolerance to salt and drought, and to decrease sensitivity to ABA (Qiu and Yu, 2009). Overexpression of *OsWRKY47* increased plant tolerance to drought and yield compared to WT (Raineri et al., 2015). This evidence demonstrated that the *WRKY* gene family may contain important regulatory factors involved in plant response to abiotic stress.

To date, genome-wide analysis has identified a large number of WRKY family members in several species with 74 *WRKY* genes in Arabidopsis (*Arabidopsis thaliana*; Ulker and Somssich, 2004), 103 in rice (*Oryza sativa* cv. Nipponbare; Ramamoorthy et al., 2008), 45 in barley (*Hordeum vulgare*; Mangelsen et al., 2008), 55 in cucumber (*Cucumis sativus*; Ling et al., 2011), 119 in maize (*Zea mays*; Wei et al., 2012), 182 in soybean (*Glycine max*; Bencke-Malato et al., 2014), and 109 in cotton (*Gossypium aridum*; Fan et al., 2015). However, there is currently no evidence regarding the *WRKY* family in the important tropical plant cassava. Cassava (*Manihot esculenta* Crantz) is the third most important crop after rice and maize in Africa, Asia, and Latin America, where it is an important food security crop (Oliveira et al., 2014). Cassava, a major staple crop, has the starchy roots that provide dietary carbohydrate for 800 million people across the tropical and sub-tropical world (International Cassava Genetic Map Consortium, 2014). Due to its high starch production and limited input, cassava is also a major producer of industrial starch and bioethanol (Zidenga et al., 2012; Perera et al., 2014). Cassava is particularly tolerant to drought and low-fertility soils when facing environmental stresses (International Cassava Genetic Map Consortium, 2014; Zeng et al., 2014). However, the mechanisms by which cassava responds to abiotic stress are poorly understood. Thus, understanding of the molecular mechanisms underlying the tolerance of cassava to abiotic stress may provide effective methods for genetic improvement of stress tolerance for cassava and other crops. The high-quality sequencing of cassava wild ancestor and cultivated varieties reported in our previous study have provided an excellent opportunity for genome-wide analysis of cassava genes (Wang et al., 2014a). Based on the significance of *WRKYs* involved in plant growth, development and adaption to the environment and on the lack of any genome-wide systematic analysis of cassava *WRKY* genes, the WRKY family was selected for a systematic analysis in cassava. In this study, 85 *WRKY* genes from the cassava genome were identified and detailed studies of their phylogeny, conserved motifs, gene structure, expression profiles in various tissues, and in response to drought, osmotic, salinity, cold, oxidative stresses, and signaling of ABA were performed. The current results may provide a novel insight into the future work on the function of WRKYs and abiotic stress responses in cassava.

## Materials and methods

### Plant materials and treatments

W14 (*M. esculenta* ssp.*flabellifolia*) is an ancestor of the wild cassava subspecies with a strong tolerance to drought stress (Wang et al., 2014a). South China 124 (SC124) is a widely planted cassava cultivar in China (Zeng et al., 2014). Argentina 7 (Arg7) adapts to a geographical high-latitude region of Argentina (Zhao et al., 2014). All plants were grown in a glass house of the Chinese Academy of Tropical Agricultural Sciences (Haikou, China). Stem segments with three nodes were cut from 8 months old cassava plants and inclined into pots with a mixture of soil and vermiculite (1:1) where they were regularly watered (Hu et al., 2015). The plants were grown from April to July 2013 during which time the temperature in the glass house ranged from 20 to 35°C. The transcripts of cassava *WRKY* genes in different tissues, including stems (90 days after planting), leaves (90 days after planting), and middle storage roots (150 days after planting) were examined with wild subspecies (W14) and cultivated variety (Arg7) under normal growth conditions. Ninety-days-old leaves and roots were sampled from Arg7, SC124 and W14 to study the transcriptional response of cassava *WRKY* genes under 12 days drought stress. After 60 days of normal cultivation, the Arg7 seedlings similar in growth vigor were used in the following treatments. For abiotic stress and signal molecule treatments, Arg7 seedlings were subjected to 200 mM mannitol for 14 days, 300 mM NaCl for 14 days, 100 μM abscisic acid (ABA) for 24 h, 3.27 M (10%) H_2_O_2_ for 24 h and low temperature (4°C) for 48 h, respectively. According to Scarpeci et al. (2013) and Ding et al. (2014), 20 mM H_2_O_2_ can induce oxidative stress in Arabidopsis. In this study, high concentration of H_2_O_2_ (3.27 M) was used to strongly induce oxidative stress due to the woody feature of cassava.

### Identification and phylogenetic analyses of the WRKY gene family in cassava

The whole protein sequence of cassava was obtained from the cassava genome database (http://www.phytozome.net/cassava.php). Sequences of the *AtWRKY* and *OsWRKY* genes were downloaded from UniPort (http://www.uniprot.org/) and RGAP databases (http://rice.plantbiology.msu.edu/), respectively. To identify the cassava WRKY family genes, two different approaches were used as follows: firstly, the local Hidden Markov Model-based searches (HMMER: http://www.ebi.ac.uk/Tools/hmmer/) built from known WRKYs to search the cassava genome database (Finn et al., 2011); secondly, BLAST analyses with all the Arabidopsis and rice WRKYs as queries were employed to check the predicted WRKYs in cassava database. With the help of CDD (http://www.ncbi.nlm.nih.gov/cdd/) and PFAM databases (http://pfam.sanger.ac.uk/), all the potential cassava WRKY genes identified from HMM and BLAST searchs were only accepted if they contained the WRKY domain, then using multiple sequence alignments to confirm the conserved domains of predicted WRKY sequences. Additionally, Clustal X 2.0 and MEGA 5.0 were used to constructed a bootstrap neighbor-joining (NJ) phylogenetic tree based on amino acid sequence of WRKY domains of cassava WRKY members and selected Arabidopsis WRKYs with 1000 bootstrap replicates (Larkin et al., 2007; Tamura et al., 2011). Furthermore, to better exhibit the characteristic of MeWRKY gene structure and conserved motifs, a NJ phylogenetic tree was created based on the full amino acids of cassava WRKYs.

### Protein properties and sequence analyses

The online ExPASy proteomics server (http://expasy.org/) was used to investigate the molecular weight (MW) and isoelectric points (pI) of presumed WRKY proteins. The conserved motifs in full-length WRKY proteins were identified using the MEME program (http://meme.nbcr.net/meme/cgi-bin/meme.cgi). Parameters employed in the analysis were: maximum number of motifs was 10 and the optimum width of motifs was set from 15 to 50 (Tao et al., 2014). Furthermore, all identified motifs were annotated according to InterProScan (http://www.ebi.ac.uk/Tools/pfa/iprscan/). The gene structures were identified by gene structure display server program (GSDS, http://gsds.cbi.pku.edu.cn/). Exon/intron organization was further checked by alignment of coding sequence and genomic DNA sequence of each *WRKY* gene.

### Transcriptomics analysis

Total RNA was extracted from stems, leaves and storage roots in Arg7 and W14 under normal growth conditions, and was also extracted from leaves and roots of Arg7, SC124 and W14 under normal conditions and 12 days drought treatment. Total RNA was isolated using plant RNeasy extraction kit (TIANGEN, China) following manufacturer's instructions and the concentration and purity were evaluated by NanoDrop 2000c (Thermo Scientific, USA). Three μg total RNA of each sample were used to construct the RNA pools according to the Illumina instructions, and subsequently sequenced by Illumina GAII following Illumina RNA-seq protocol. A total of 610.70 million 51-bp raw reads was generated from the 18 samples. Adapter sequences were removed from raw sequence reads using FASTX-toolkit (version 0.0.13, http://hannonlab.cshl.edu/fastx_toolkit/). Sequence quality was examined using FastQC (http://www.bioinformatics.babraham.ac.uk/projects/fastqc/) and low quality sequences (including reads with unknown base pairs “N”) were removed, which produced 583.82 million clean reads. Clean reads were mapped to cassava reference genome (version 4.1) derived from the phytozome website (ftp://ftp.jgi-psf.org/pub/compgen/phytozome/v9.0/Mesculenta/) using Tophat v.2.0.10 (http://tophat.cbcb.umd.edu/) (Trapnell et al., 2009), and 88.7% reads were aligned. The resulting alignment files are provided as input for Cufflinks to generate transcriptome assemblies (Trapnell et al., 2012). Gene expression levels were calculated as FPKM according to the length of the gene and reads count mapped to this gene: FPKM = total exon fragments/[mapped reads (millions) × exon length (kb)]. DEGseq was applied to identify differentially expressed genes with a random sampling model based on the read count for each gene (Wang et al., 2010).

### Quantitative RT-PCR analysis

Expression of *MeWRKY* genes in response to various abiotic stress (osmotic, salt, cold, and oxidative stress) and ABA signaling were examined by qRT-PCR analysis with Stratagene Mx3000P Real-Time PCR system (Stratagene, CA, USA) using SYBR® Premix Ex Taq™ (TaKaRa, Japan) according to the manufacturer's instructions. Total RNA was extracted from leaves of control and treated samples. Two hundred ng Poly(A)^+^ mRNA from each treatment was converted into cDNA using AMV Reverse Transcriptase (Promega, Madison, WI, USA) at 42°C in a 20 μL reaction volume that subsequently served as the template for qRT-PCR. The amplification conditions used for all PCRs were implemented as follows: 10 min at 95°C, and followed by 40 cycles of 10 s at 95°C, 15 s at 50°C, and 30 s at 72°C. The relative expression of the target genes was determined using the 2^−ΔΔCt^ method (Livak and Schmittgen, 2001). The specific primers were designed according to the *WRKY* gene sequences by Primer 5.0 software (Table S1). Subsequently, reaction specificities for each primer pair was tested using qRT-PCR melting curve analysis, agarose gel electrophoresis, and sequencing PCR products. Amplification efficiencies of gene-specific primers ranged from 90 to 110%. β-tubulin gene (TUB) and elongation factors 1α gene (EF1) verified to be constitutive expression and suitable as internal controls were used as internal references for all the qRT-PCR analyses (Salcedo et al., 2014). Each treated sample contained a corresponding regularly-watered control and each sample was performed with three independent biological replications. Then, the treated and control plants at each time point were sampled to perform expression analysis. The relative expression levels of *MeWRKY* genes in each treated time point were compared with corresponding regularly-watered control (Wang et al., 2014b). Statistical difference were performed by Duncan's multiple range test (*n* = 3). Means denoted by the same letter do not significantly differ at *P* < 0.05.

## Results and discussion

### Identification and phylogenetic analysis of cassava WRKYs

To identify the WRKY family members in cassava, both BLAST and HMMER searches were performed to search the cassava genome with Arabidopsis and rice WRKY sequences as queries. After these searches, a total of 85 putative members of the WRKY family were detected in the complete cassava genome. Conserved domain analysis further confirmed that all the WRKYs contain single or double WRKY domains at the N-terminus, which are the basic characteristics of WRKY family. The 85 predicted WRKY proteins ranged from 149 (MeWRKY22) to 737 (MeWRKY64) amino acids (aa) in length with an average of 369.4 aa, the relative molecular mass varied from 17.19 kDa (MeWRKY22) to 79.76 kDa (MeWRKY64), and the pIs ranged from 4.91 (MeWRKY59) to 9.89 (MeWRKY1) with 38 numbers pI >7 and others pI <7 (Table S2). cDNAs of all 85 *MeWRKY* genes have been submitted to GenBank and their accession numbers in GenBank are shown in Table S3.

**Figure 1:**
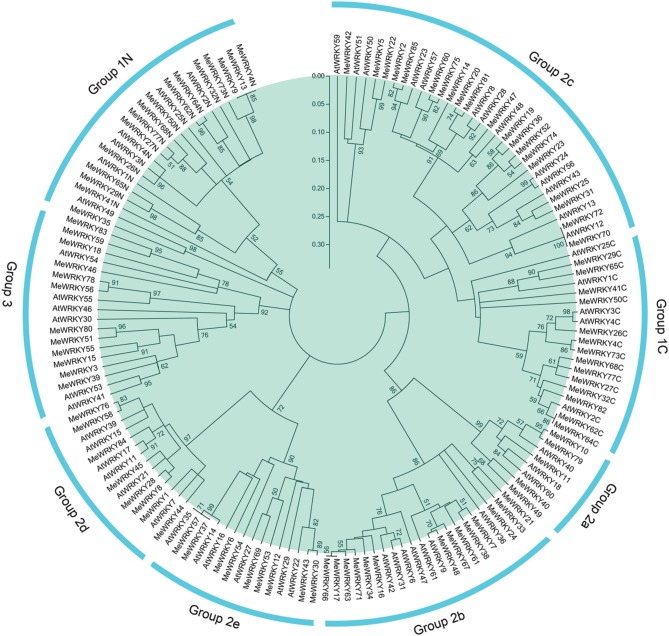
**Phylogenetic analysis of WRKY proteins from cassava and Arabidopsis**. The NJ tree was constructed with WRKY domains of WRKYs from cassava and Arabidopsis using ClustalX 2.0 and MEGA5 with 1000 bootstrap. Branches with less than 50% bootstrap support were collapsed. The WRKY proteins are grouped into three groups (1–3) and five subgroups (2a–2e). Group 1 proteins with the suffix “N” or “C” indicates the N-terminal WRKY domains or the C-terminal WRKY domains. “AtWRKYs” are the WRKY proteins from Arabidopsis. “MeWRYKs” indicate the WRKY proteins from cassava.

To study the evolutionary relationships between cassava WRKY proteins and known WRKYs from Arabidopsis, an unrooted neighbor-joining phylogenetic tree was created based on multiple alignments of the predicted amino acid sequences of the WRKY domains from cassava and Arabidopsis. As shown in Figure 1, 85 MeWRKY proteins were classified into three major groups, among which group 2 was subdivided into five subgroups together with WRKYs from Arabidopsis. This was in accordance with the classification of WRKY family in Arabidopsis (Eulgem et al., 2000), cucumber (Ling et al., 2011), maize (Wei et al., 2012), and soybean (Bencke-Malato et al., 2014). Groups 1, 2, and 3 contained 17, 56, and 12 MeWRKY proteins, respectively. A total of 5, 14, 20, 8, and 9 proteins were assigned to subgroups 2a, 2b, 2c, 2d, and 2e, respectively. Generally, group 1 contained two WRKY domains, but there were a few MeWRKY proteins that contained only one WRKY domain, such as, MeWRKY9, -13, -35, and -82. The same phenomenon was also found in Arabidopsis (Eulgem et al., 2000) and maize (Wei et al., 2012). According to one previous report (Wei et al., 2012), the loss of WRKY domain seems to be more common in monocotyledons than in dicotyledons. It can be deduced that group 1 might contain the original genes of other groups and that MeWRKY9, -13, -35, and -82 emerged later during evolution.

Phylogenetic analysis also showed that there were some closely related orthologous WRKYs between cassava and Arabidopsis (MeWRKY42 and AtWRKY51; MeWRKY47 and AtWRKY48; MeWRKY48 and AtWRKY9; MeWRKY69 and AtWRKY27; MeWRKY35 and AtWRKY49; MeWRKY70/MeWRKY72 and AtWRKY12; MeWRKY37/MeWRKY57 and AtWRKY35; MeWRKY1/MeWRKY44 and AtWRKY7; MeWRKY45 and AtWRKY21; MeWRKY26 and AtWRKY3/AtWRKY4), suggesting that an ancestral set of WRKY genes existed prior to the divergence of cassava and Arabidopsis and that WRKYs from cassava generally have close relationship with the proteins from Arabidopsis. MeWRKY1 and MeWRKY44 showed a high degree of similarity with AtWRKY7, which was reported to negatively regulate plant defense against bacterial pathogens (Kim et al., 2006). MeWRKY69 shared considerable similarity with AtWRKY27 that is also involved in the regulation of plant defense against the bacterial pathogens by regulating the expression of nitrogen metabolism and nitric oxide (NO) generation genes (Mukhtar et al., 2008). AtWRKY51, which showed a high degree of similarity with MeWRKY42, was reported to mediate jasmonic acid (JA) signaling and partially alter resistance to virulent pathogens (Gao et al., 2011). These results suggested the possible functions of WRKY genes in cassava.

### Conserved motifs and gene structure of cassava WRKYs

**Figure 2:**
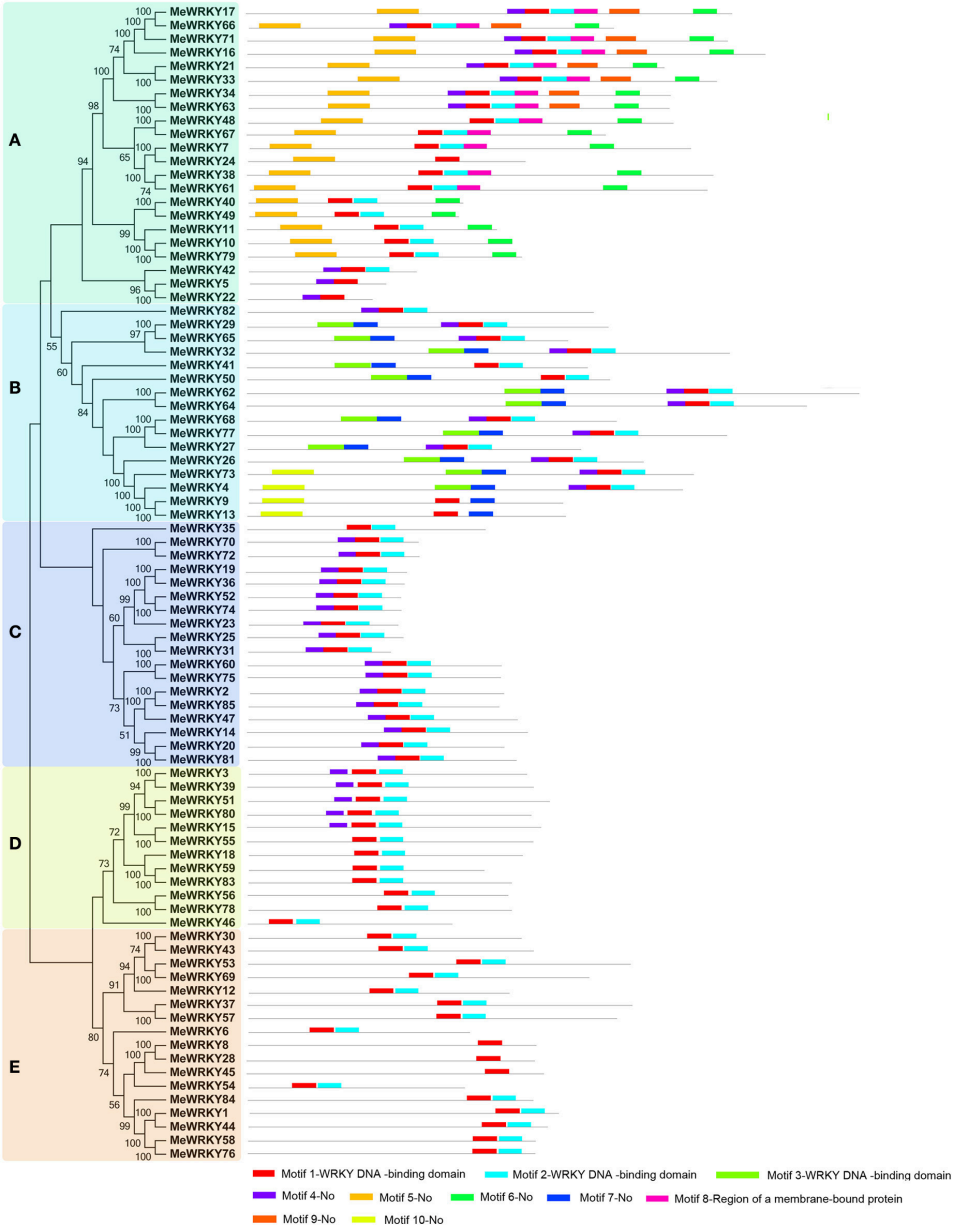
**Conserved motifs of MeWRKY proteins according to the phylogenetic relationship**. The NJ tree was constructed with full amino acids of cassava WRKYs using ClustalX 2.0 and MEGA5 with 1000 bootstraps. The conserved motifs in the MeWRKY proteins were identified by MEME. Gray lines represent the non-conserved sequences, and each motif is indicated by a colored box numbered at the bottom. The length of motifs in each protein was exhibited proportionally. **(A–E)** indicates different groups of WRKY family in cassava.

To further detect the structural features of cassava WRKYs, conserved motifs and intron/exon distribution were analyzed according to their phylogenetic relationships. A total of 10 conserved motifs in cassava WRKYs were found using MEME software and further annotated by InterPro Scan 5 (Figure 2; Figure S1). Results showed that three (1–3) of 10 motifs were annotated as WRKY DNA-binding, which is a basic characteristics of the WRKY family. All MeWRKYs contained at least one of them, indicating that the cassava WRKYs identified in this study had conserved features of the WRKY family. Notably, all the MeWRKYs contain at least two motifs, except for three members (MeWRKY8, -28, and -45) only containing motif 2 in cluster E. In cluster A, all the MeWRKYs, except for MeWRKY5, -22, -24, and -42, contained motifs 1, 2, 6, and 9. Interestingly, most of the MeWRKY members in cluster A specially showed motifs 8 and 9 in comparison to MeWRKYs in other clusters. In cluster B, all the MeWRKYs, except for MeWRKY82, contained motifs 1 and 7, and motif 10 was uniquely dispersed in four members (MeWRKY4, -9, -13, and -73). In cluster C, all members contained motifs 1, 2, and 4, except for MeWRKY35 which did not contain the motif 4. In cluster D, all members contained motifs 1 and 2, and five members (MeWRKY3, -39, -51, -80, and -15) also contained motif 4 in addition to motifs 1 and 2. In cluster E, all members contained motifs 1 and 2, except for the closely related MeWRKY8, -28, and -45, which only contained motif 1. Generally, WRKY members in the same cluster commonly shared similar motif compositions, indicating functional similarity among them.

**Figure 3:**
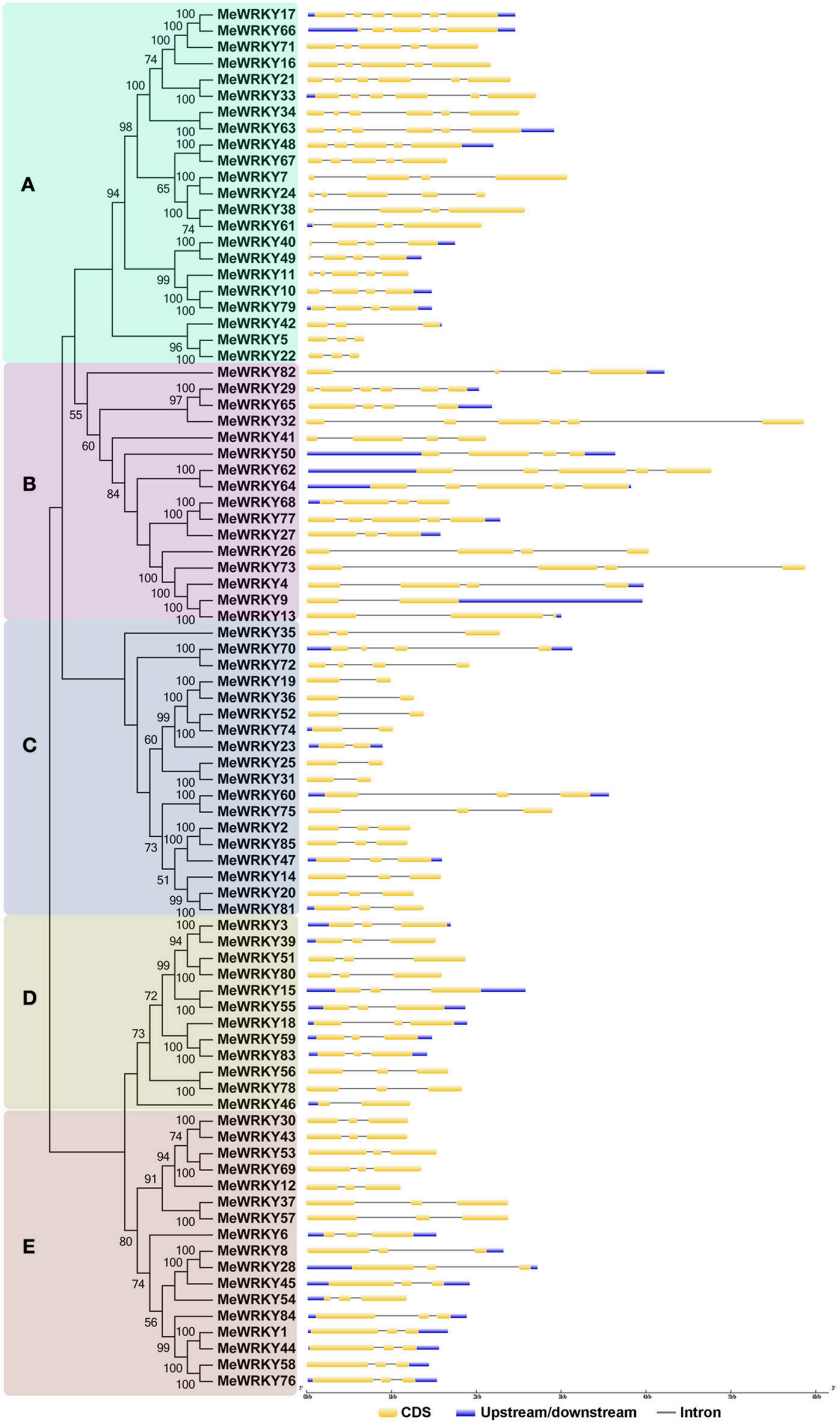
**The exon-intron structure of ***MeWRKY*** genes according to the phylogenetic relationship**. The unrooted phylogenetic tree was constructed based on the full length sequences of MeWRKYs with 1000 bootstraps. Exon-intron structure analyses of *MeWRKY* genes were performed by using the online tool GSDS. Lengths of exons and introns of each *MeWRKY* gene were exhibited proportionally. **(A–E)** indicates different groups of WRKY family in cassava.

Exon-intron structural diversity, an important part in the evolution of gene families, provides additional evidence supporting phylogenetic groupings (Shiu and Bleecker, 2003; Wang et al., 2014c). Intron/exon distribution was analyzed to better understand phylogenetic relationship and classification of cassava WRKYs. As shown in Figure 3, the number of introns in *MeWRKY* genes varied from 1 (*MeWRKY9*, -*19, -23*, -*25*, -*31*, -*36*, -*46*, -*52*, and -*74*) to 5 (*-17*, -*21*, -*29*, -*32*, -*33*, -*34*, and -*63*). However, in rice and rubber tree, the number of introns varied from 0 (*OsWRKY10* and *OsWRKY44*) to 8 (*OsWRKY41.D1* and *OsWRKY41.D2*) and 1 (*HbWRKY22, -34*, -*35* and -*36*) to 7 (*HbWRKY15*), respectively (Xie et al., 2005; Li et al., 2014). These results indicated that *WRKYs* in cassava have less gene structure diversity than that in rice and rubber tree. Additionally, 42 out of 85 *MeWRKY* genes each had two introns. The same phenomenon was also observed in rice and rubber tree with 42 of 92 and 40 of 81 *WRKY* genes containing two introns each, respectively (Xie et al., 2005; Li et al., 2014). Cluster A contained 2–5 introns; cluster B contained 1–5 introns; cluster C contained 1–3 introns; all cluster D *MeWRKYs* contained 2 introns, except for *MeWRKY46* with only one intron; and cluster E *MeWRKYs* contained two introns. According to a previous report (Nuruzzaman et al., 2010), the rate of intron loss is faster than the rate of intron gain after segmental duplication in rice. Consequently, it can be concluded that clusters A and B might contain the original genes, from which those in other clusters were derived. Generally, *MeWRKYs* in the same cluster of the phylogenetic tree show similar exon-intron structures.

### Expression profiles of *MeWRKY* genes in different tissues

**Figure 4:**
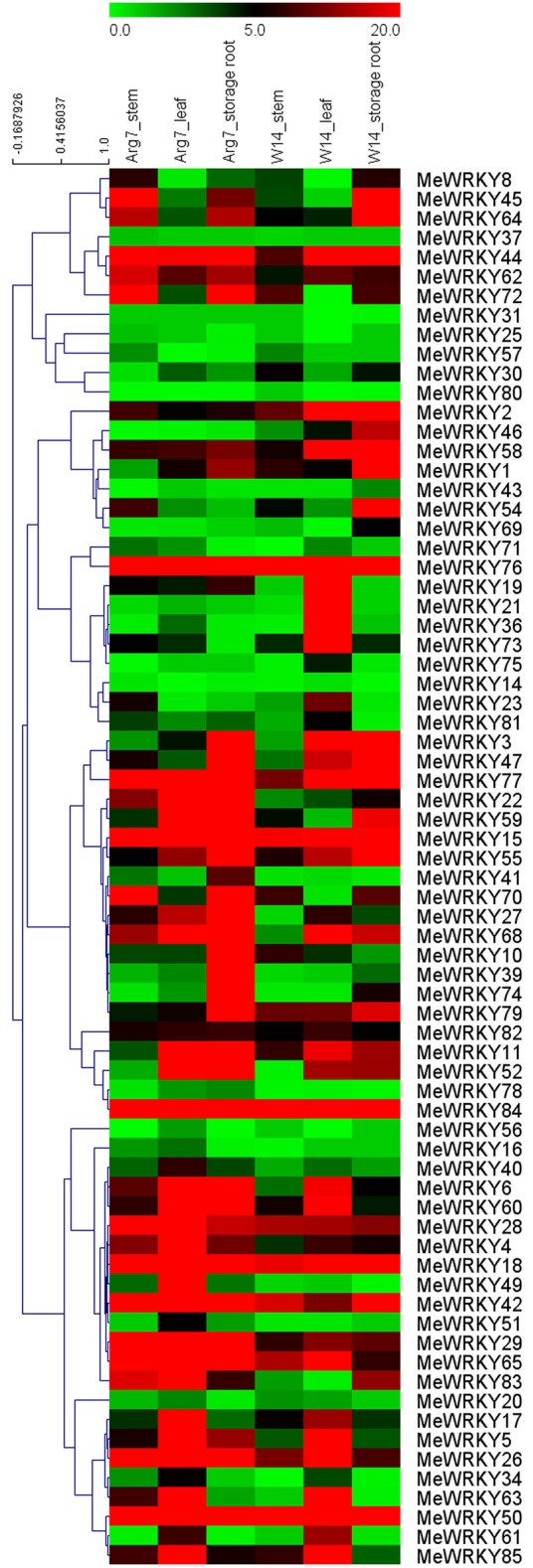
**Expression profiles of ***MeWRKY*** genes in different tissues of two cassava accessions**. FPKM value was used to create the heat map with clustering. The scale represents the relative signal intensity of FPKM values.

To provide some clues on the roles of *MeWRKY* genes in cassava growth and development, the expression profiles of *MeWRKY* genes from different organs, including stems, leaves and storage roots were tested in a wild subspecies (W14) and cultivated variety (Arg7) using transcriptomic data. W14, a wild cassava subspecies, has a low rate of photosynthesis, tuber root yield, and starch content in root tubers, but strong tolerance to drought stress (Wang et al., 2014a). Arg7, a cultivated variety, can tolerate moderate drought stress (Zhao et al., 2014). Expression analysis of *MeWRKY* genes in these two accessions will provide insight into cassava development between wild subspecies and cultivated variety. Seventy-two of 85 *MeWRKY* genes were captured from the corresponding transcriptomic data (Figure 4; Table S4).

In the Arg7 variety, 100% (72/72), 94.4% (68/72), and 91.7% (66/72) of *MeWRKY* genes were expressed in stems, leaves, and storage roots, respectively, with 50% (36/72), 55.9% (38/68), and 63.6% (42/66) of *MeWRKY* genes showing high expression levels (value >5) in stems, leaves, and storage roots, respectively. Moreover, 90.3% (65/72) of *MeWRKY* genes were expressed in all organs examined, among which 40% (26/65) showed high expression levels (value >5) in all three organs.

In the W14 subspecies, 91.7% (66/72), 98.6% (71/72), and 88.9% (64/72) of *MeWRKY* genes were found to be expressed in stems, leaves, and storage roots, respectively, with 40.9% (27/66), 54.9% (39/71), and 57.8% (37/64) of *MeWRKY* genes showing high expression levels (value >5) in stems, leaves, and storage roots, respectively. Moreover, 81.9% (59/72) of *MeWRKY* genes were expressed in all organs examined, among which 30.5% (18/59) showed high expression levels (value >5) in all three organs.

About 20.8% (15/72) of *MeWRKY* genes with high expression levels (value >5) in all three tested organs in Arg7 and W14, suggesting that *MeWRKY* genes may be involved in organ development. Transcriptomic data also showed that 56 *MeWRKY* genes had a constitutive expression pattern that expressed in all the tissues of the two accessions, suggesting that these genes might play a role in plant growth, development, and cellular homeostasis. The remaining 16 *MeWRKY* genes exhibited differential expression patterns, with specific to some particular tissues, such as *MeWRKY16, MeWRKY20*, and *MeWRKY23*. This phenomenon was also observed in rice (Ramamoorthy et al., 2008), cucumber (Ling et al., 2011), rubber tree (Li et al., 2014) and grape (Wang et al., 2014c), indicating that the functions of the WRKYs are diverse in both monocotyledon and dicotyledon.

There were 33 *MeWRKY* genes that showed higher expression levels in leaf and stem tissues in Arg7 than that in W14. However, 25 *MeWRKY* genes had higher expression levels in storage roots in W14 than that in Arg7. Interestingly, *MeWRKY8, -18, -34, -45, -54, -80*, and *-83* showed higher expression levels in Arg7 than in W14 in leaf and stem tissues, but opposite result was observed in storage roots. These *MeWRKY* genes have strong expression levels for special tissues in different accessions, indicating their key roles in tissue development or tissue functions.

Generally, 15 out of 72 *MeWRKY* genes had high transcript abundance (value >5) in all the tested tissues of the two accessions, including *MeWRKY16, -29, -50, -65*, and *-77* in group 1, *MeWRKY2*, and *-42* in group 2c, *MeWRKY28, -44, -58, -76*, and *-84* in group 2d and *MeWRKY15, -18*, and *-55* in group 3. In contrast, 3 *MeWRKY* genes (*MeWRKY14*, and *-31* in group 2c, *MeWRKY80* in group 3) showed low expression levels in all the tissues of the two accessions. Overall, the tissue expression profiles of *WRKY* genes in different accessions may lay a foundation for further investigation of cassava development.

### Expression of *MeWRKY* genes in response to drought in different accessions

**Figure 5:**
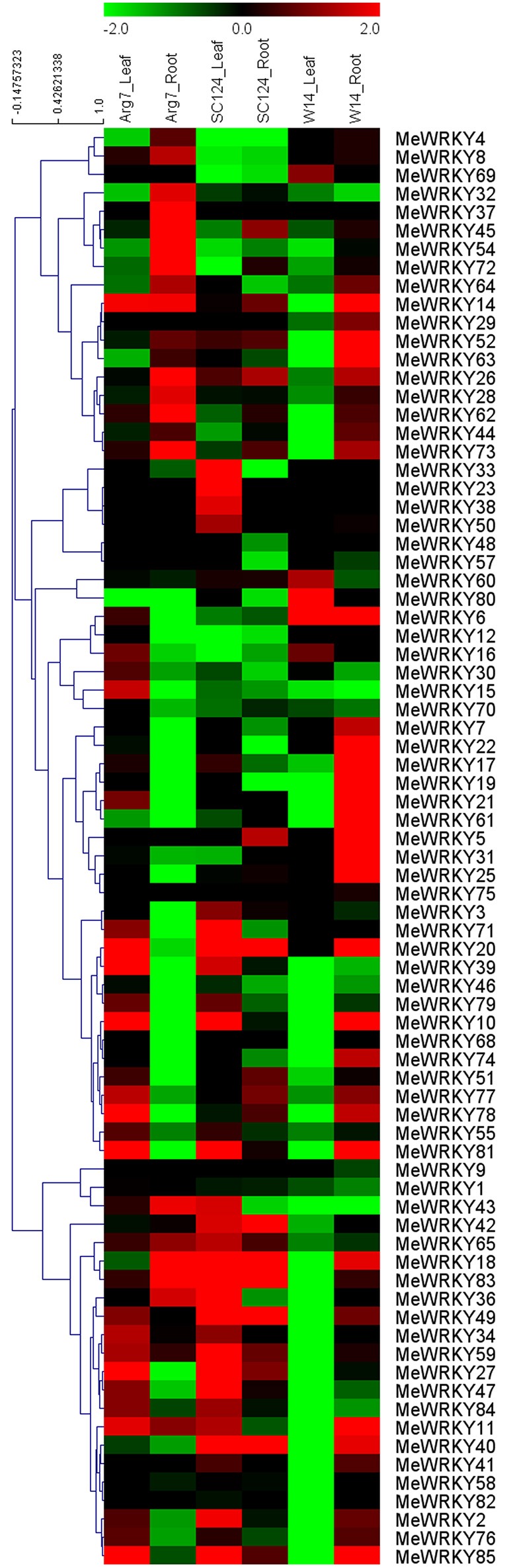
**Expression profiles of ***MeWRKY*** genes in leaves and roots of three cassava accessions after drought treatment**. Log2 based FPKM value was used to create the heat map with clustering. The scale represents the relative signal intensity of FPKM values.

Accumulated evidence has suggested that *WRKY* family genes play a significant role in plants' response to drought or osmotic stress (Ramamoorthy et al., 2008; Ren et al., 2010; Rushton et al., 2010; Ling et al., 2011; Tripathi et al., 2014). Thus, there is need to examine the expression patterns of *WRKY* genes in response to drought stress, which may provide important clues for further understanding the mechanisms of cassava involved in strong tolerance. For this reason, 3-month-old cassava seedlings (a wild subspecies W14 and two cultivated varieties Arg7 and SC124) were deprived of water for 12 days, and then the leaf and root tissues were collected to extract RNA for subsequent RNA-seq analysis. Heatmap representation of expression profiles of 78 *MeWRKY* genes under drought stress conditions were captured from the corresponding transcriptomic data (Figure 5; Table S5).

In the Arg7 variety, transcripts of 43.6% (34/78) and 33.3% (26/78) of *MeWRKY* genes increased after drought stress in leaves and roots, respectively, and 25.6% (20/78) and 50% (39/78) decreased in leaves and roots, respectively. Significant induction (value >1) of 21.8% (17/78) and 23.1% (18/78) of *MeWRKY* genes was observed after drought stress in leaves and roots, respectively. Eleven genes (14.1%) were upregulated in both leaves and roots, with two genes (*MeWRKY11* and *MeWRKY14*) showing significant induction (value >1).

In the SC124 variety, transcripts of 47.4% (37/78) and 33.3% (26/78) of *MeWRKY* genes increased after drought stress in leaves and roots, respectively, and 30.8% (24/78) and 50% (39/78) decreased in leaves and roots, respectively. Significant induction (value >1) of 33.3% (26/78) and 11.5% (9/78) of *MeWRKY* genes was observed after drought stress in leaves and roots, respectively. Eighteen genes (23.1%) were upregulated in both leaves and roots, with six genes (*MeWRKY18*, -*20*, -*40*, -*42*, -*49*, and -*83*) showing significant induction (value >1).

In the W14 subspecies, transcripts of 6.4% (5/78) and 55.1% (43/78) of *MeWRKY* genes increased after drought stress in leaves and roots, respectively, and 67.9% (53/78) and 24.4% (19/78) decreased in leaves and roots, respectively. Significant induction (value >1) of 5.1% (4/78) and 32.1% (25/78) of *MeWRKY* genes was observed after drought stress in leaves and roots, respectively. Only *MeWRKY6* was upregulated in both leaves and roots.

The transcriptomic data given above showed that there were significantly more *WRKY* genes upregulated by drought at the transcription level in roots than in leaves in W14, but there were fewer in roots than in leaves in Arg7 and SC124. There were also more *WRKY* genes significantly induced by drought (value >1) in roots than in leaves in W14 but fewer in roots than in leaves in SC124. W14 showed stronger tolerance to drought stress than SC124 and Arg7, two varieties commonly cultivated in China and Southeast Asia, respectively (Wang et al., 2014a). Cassava can form deep root systems (soil depth below 2 m), which is beneficial for penetrating into deeper soil layers and absorbing water stored in the soil (Okogbenin et al., 2013). Moreover, numerous studies have confirmed that the *WRKY* family genes play a positive role in the drought stress response in various species (Qiu and Yu, 2009; Ren et al., 2010; Jiang et al., 2012; Ding et al., 2014; Raineri et al., 2015). Therefore, these findings indicate that cassava *WRKY* genes might play an important role in water uptake from soil by roots, and hence maintaining strong tolerance to drought stress in W14 subspecies.

Generally, *MeWRKY* genes showed similar expression profiles in leaves or roots tissues in Arg7 and SC124, which was different from W14. After drought treatment, expression of some *MeWRKY* genes, including *MeWRKY2*, -*6*, -*7*, -*10*, -*17*, -*19*, -*22*, -*31*, -*74*, and -*76*, were upregulated in roots of W14, but downregulated in roots of SC124 and Arg7. The transcripts of some *MeWRKY* genes, including *MeWRKY2*, -*10*, -*11*, -*14*, -*17*, -*27*, -*34*, -*39*, -*43*, -*47*, -*49*, -*55*, -*59*, -*65*, -*76*, -*77*, -*81*, -*83*, -*84*, and -*85*, increased in leaves of Arg7 and SC124, but decreased in leaves of W14 after drought treatment. *WRKY* genes in different accessions showed different expression profiles in response to drought, suggesting that the mechanisms of *WRKYs* involved in drought response differ between wild subspecies and cultivated varieties. Additionally, although some *MeWRKY* genes showed close phylogenetic relationships, their transcriptional levels showed different responses to drought, such as, *MeWRKY4* and -*9, MeWRKY27* and -*77, MeWRKY29* and -*65*, and *MeWRKY50* and -*68* in group 1, *MeWRKY21* and -*33* in group 2b, and *MeWRKY70* and -*72* in group 2c. Taken together, the transcriptional response of *MeWRKY* genes to drought stress in wild subspecies and cultivated varieties may provide an opportunity for further investigation of the mechanisms underlying strong drought tolerance in cassava.

### Temporal expression profiles of *MeWRKY* genes upon exposure to various stress and related signaling

*WRKY* genes have been reported to play pivotal role in the regulation of plant tolerance to various stress and related signaling transduction in various species (Rushton et al., 2010, 2012; Tripathi et al., 2014; Banerjee and Roychoudhury, 2015). Hence, to investigate the roles of *MeWRKY* genes in response to various environmental stresses and related signaling, the expression profiles of *MeWRKY* genes under these treatments were analyzed. Nine *MeWRKY* genes (*MeWRKY8*, -*28*, -*37*, -*49*, -*62*, -*65*, -*76*, -*83*, and -*85*) distributed in different subgroups and up-regulated by drought stress as indicated by RNA-seq data in different cassava accessions were selected for further examination of their transcriptional response to osmotic, salt, cold, ABA, and H_2_O_2_ treatments.

**Figure 6:**
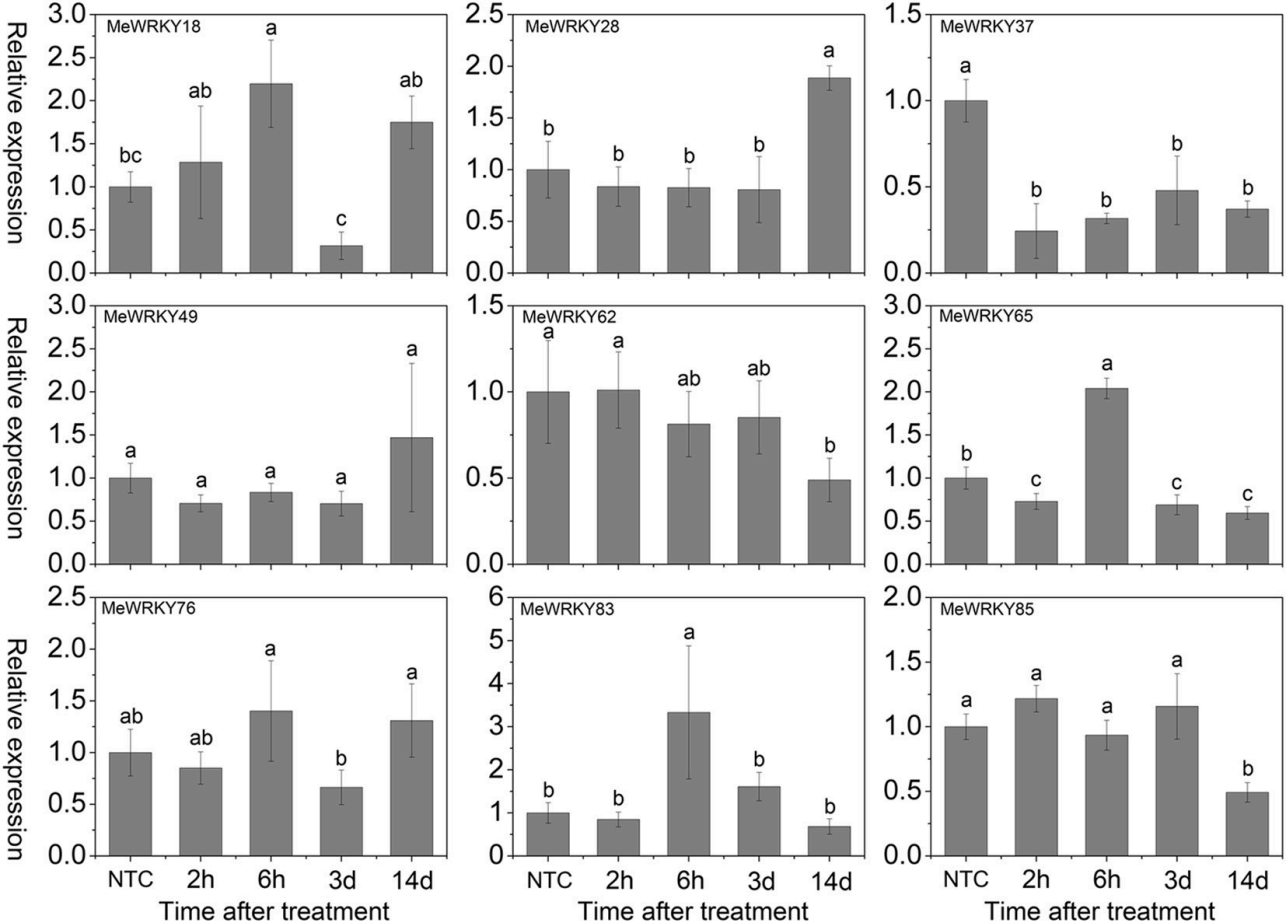
**Expression profiles of ***MeWRKY*** genes in leaves under salt stress**. The relative expression levels of *MeWRKY* genes in each treated time point were compared with that in each time point at normal conditions. NTC (no treatment control) at each time point was normalized as “1.” Data are means ± SE calculated from three biological replicates. Values with the same letter were not significantly different according to Duncan's multiple range tests (*P* < 0.05, *n* = 3).

Under NaCl treatment, *MeWRKY18* was induced after 2-6 h and 14 days treatment with significant up-regulation at 6 h. *MeWRKY65* and *MeWRKY83* were significantly induced at 6 h. *MeWRKY28* was significantly induced at 14 days, while *MeWRKY85* was visibly down-regulated at 14 days. *MeWRKY37* showed down-regulation at all the treated time-points. Other three *WRKY* genes (*MeWRKY49*, -*62*, and -*76*) did not display obvious trends during salt treatment (Figure 6). In Arabidopsis, some *WRKY* genes, including *AtWRKY8* (Hu et al., 2013), *AtWRKY18* (Chen et al., 2010), *AtWRKY25* (Jiang and Deyholos, 2009), *AtWRKY30* (Scarpeci et al., 2013), *AtWRKY33* (Jiang and Deyholos, 2009), *AtWRKY40* (Chen et al., 2010), *AtWRKY46* (Ding et al., 2014), *AtWRKY60* (Chen et al., 2010), and *AtWRKY75* (Yu et al., 2010), were reported to be up-regulated at transcriptional levels after salt treatment. Similarly, about 26 rice *WRKY* genes showed up-regulation upon salt stress treatment (Ramamoorthy et al., 2008; Yu et al., 2010; Tao et al., 2011). Accumulating evidence has suggested that some *WRKY* genes play a positive role of in response to salt stress, such as *AtWRKY8* (Hu et al., 2013), *AtWRKY25* (Jiang and Deyholos, 2009), *AtWRKY30* (Scarpeci et al., 2013), and *AtWRKY33* (Jiang and Deyholos, 2009). However, other *WRKYs*, including *AtWRKY18* (Chen et al., 2010), *OsWRKY45-2* (Tao et al., 2011), *AtWRKY46* (Ding et al., 2014), and *AtWRKY60* (Chen et al., 2010) were found to act as negative regulators in salt stress response in Arabidopsis and rice. These studies indicated that *MeWRKY* genes may be involved in the salt stress response.

**Figure 7:**
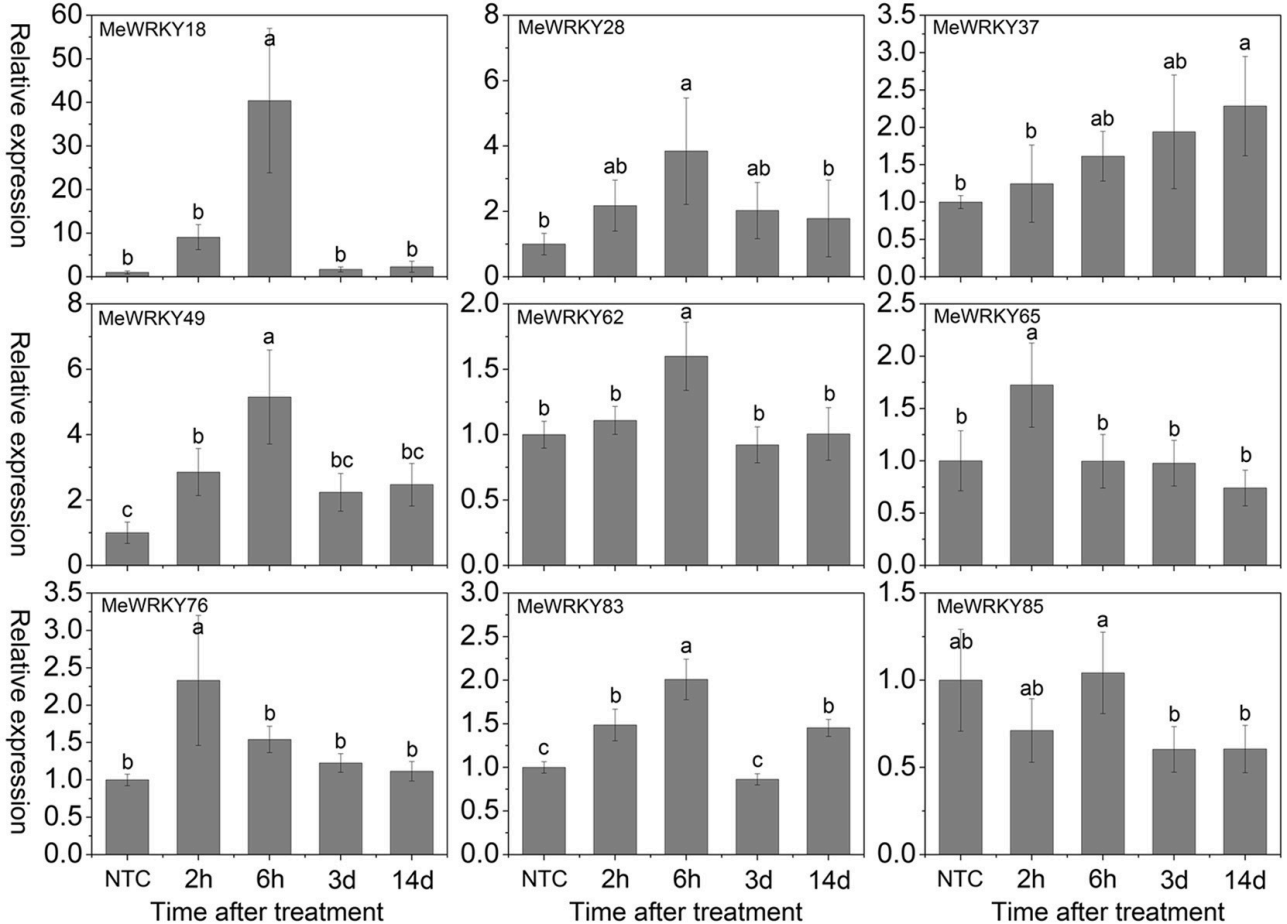
**Expression profiles of ***MeWRKY*** genes in leaves under osmotic stress**. The relative expression levels of *MeWRKY* genes in each treated time point were compared with that in each time point at normal conditions. NTC (no treatment control) at each time point was normalized as “1.” Data are means ± SE calculated from three biological replicates. Values with the same letter were not significantly different according to Duncan's multiple range tests (*P* < 0.05, *n* = 3).

As shown in Figure 7, under mannitol treatment, *MeWRKY18*, -*28*, -*37*, -*49*, and -*76* were induced during 2 h 14days treatment and showed significant induction at 6 h, 6 h, 14 days, 6 h, and 2 h, respectively. *MeWRKY62* and *MeWRKY83* expression were induced during 2–6 h and 14 days treatment with significant up-regulation at 6 h. *MeWRKY65* showed induction at 2 h treatment. *MeWRKY85* did not show obvious trends during mannitol treatment. Notably, *MeWRKY18* showed up-regulation at all treated points and reached the highest expression level (value >40) at 6 h, indicating its possible roles in osmotic/drought stress responses. In Arabidopsis, some *WRKY* genes, including *AtWRKY57* (Jiang et al., 2012) and *AtWRKY63/ABO3* (Ren et al., 2010), have been reported to positively regulate drought stress tolerance. However, some *WRKY* genes, including *AtWRKY18* (Chen et al., 2010), *AtWRKY46* (Ding et al., 2014), *AtWRKY53* (Sun and Yu, 2015), *AtWRKY54* (Li et al., 2013), *AtWRKY60* (Chen et al., 2010), and *AtWRKY70* (Li et al., 2013), which showed significant induction during drought stress, have been reported to negatively regulate drought stress tolerance. *MeWRKY18*, showing high similarity with *AtWRKY54*, may represent a functional gene involved in drought tolerance in cassava. In rice, 23 *WRKY* genes have been reported to be induced under drought treatment (Ramamoorthy et al., 2008; Qiu and Yu, 2009; Wu et al., 2009; Shen et al., 2012; Raineri et al., 2015), among which *OsWRKY11* (Wu et al., 2009), *OsWRKY30* (Shen et al., 2012), *OsWRKY45* (Qiu and Yu, 2009), and *OsWRKY47* (Raineri et al., 2015) have been confirmed to function as positive factors in the regulation of plant tolerance to drought/osmotic stress. In cucumber, the expression of 4 *WRKY* genes (*CsWRKY2*, -*14*, -*18*, -*21*) was found to be upregulated after drought treatment (Ling et al., 2011). In cotton (*Gossypium hirsutum*) roots, 15 out of 26 *GhWRKs* (*GhWRKY9*, -*10*, -*11*, -*13*, -*14*, -*17*, -18, -19, -*20*, -*23*, -*24*, -*29*, -*32*, -*33*, and -*34*) and 7 out of 26 *GhWRKs* (*GhWRKY12*, -15, -21, -22, -26, -27, and -30) were up- and down-regulated, respectively, under dehydration conditions (Zhou et al., 2014). Together, these results indicate the important roles of these *WRKY* genes in response to osmotic/drought stress.

**Figure 8:**
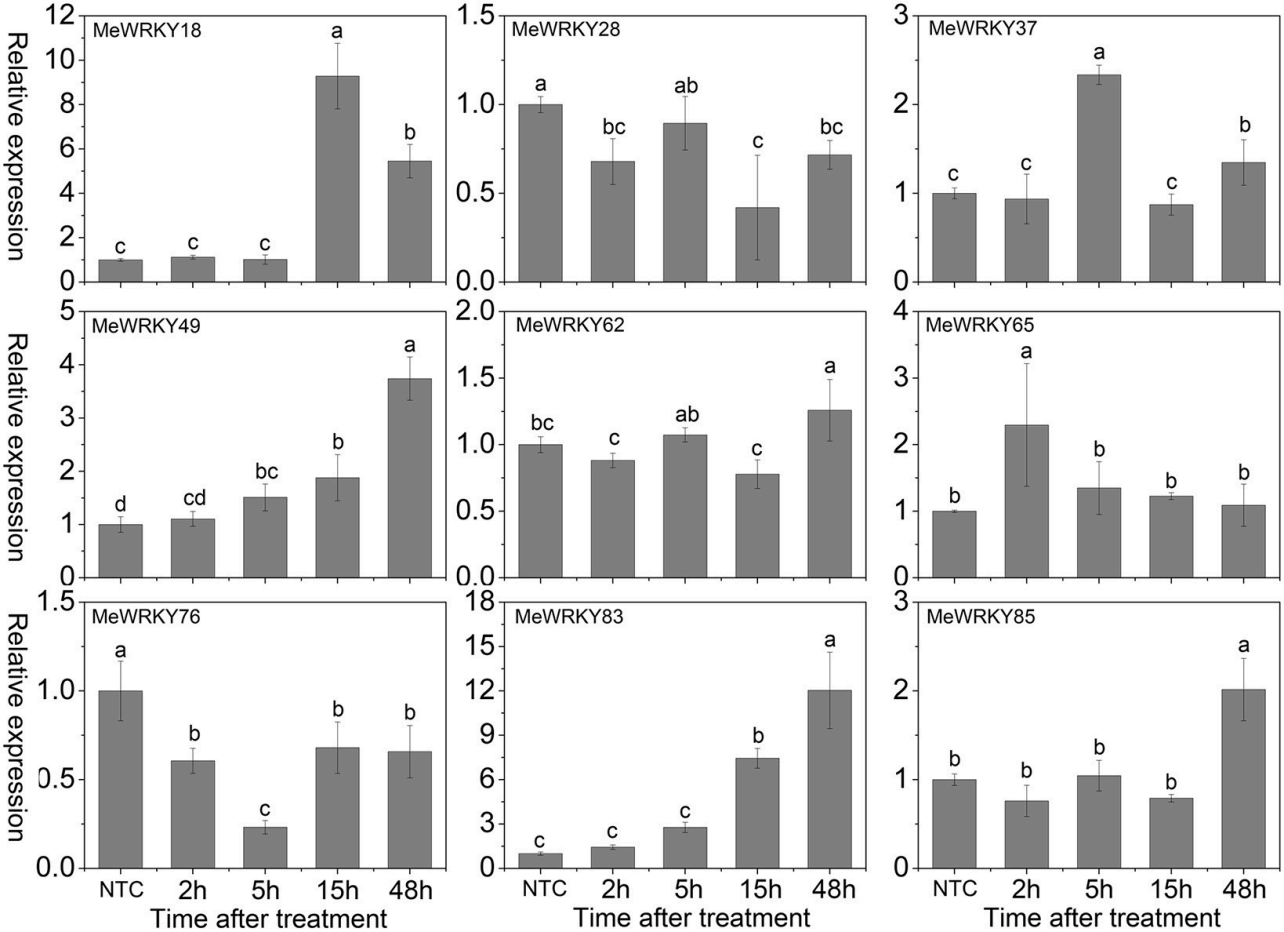
**Expression profiles of ***MeWRKY*** genes in leaves under cold treatment**. The relative expression levels of *MeWRKY* genes in each treated time point were compared with that in each time point at normal conditions. NTC (no treatment control) at each time point was normalized as “1.” Data are means ± SE calculated from three biological replicates. Values with the same letter were not significantly different according to Duncan's multiple range tests (*P* < 0.05, *n* = 3).

Cold stress, a common environmental stress, affects plants growth and crop productivity, especially in tropical and sub-tropical origin (Wang et al., 2012). However, little is known about the mechanisms underlying the action of WRKYs in cold stress response. In Arabidopsis, *WRKY34* was reported to be significantly induced by cold treatment and act as a negative regulator to cold response (Zou et al., 2010). In rice, 2 and 15 *WRKY* genes were up- and down-regulated by cold treatment, respectively (Ramamoorthy et al., 2008; Yokotani et al., 2013). Among them, overexpression of *OsWRKY76* increased tolerance to cold stress (Yokotani et al., 2013). Under cold treatment, *MeWRKY18*, -*49*, -*65*, and -*83* showed up-regulation at all the treated time-points, with significant up-regulation at 15, 48, 2, and 48 h, respectively. *MeWRKY37*, -*62*, and -*85* showed significant up-regulation at 5, 48, and 48 h, respectively. However, *MeWRKY28* and -*76* expression was repressed during all the treated time points (Figure 8). The expression levels of *MeWRKY49* and -*83* increased as treatment time continued, suggesting their possible function in cold response. They could be used in further functional characterization. Cassava, an important tropical crop, is distributed in tropical areas all over the world. Cold stress significantly restricts plant growth, agricultural productivity, and the development of cassava. Research on *WRKY*-mediated cold response in cassava may benefit further functional characterization of *WRKY* genes and investigations of the mechanisms underlying the cold response in cassava.

**Figure 9:**
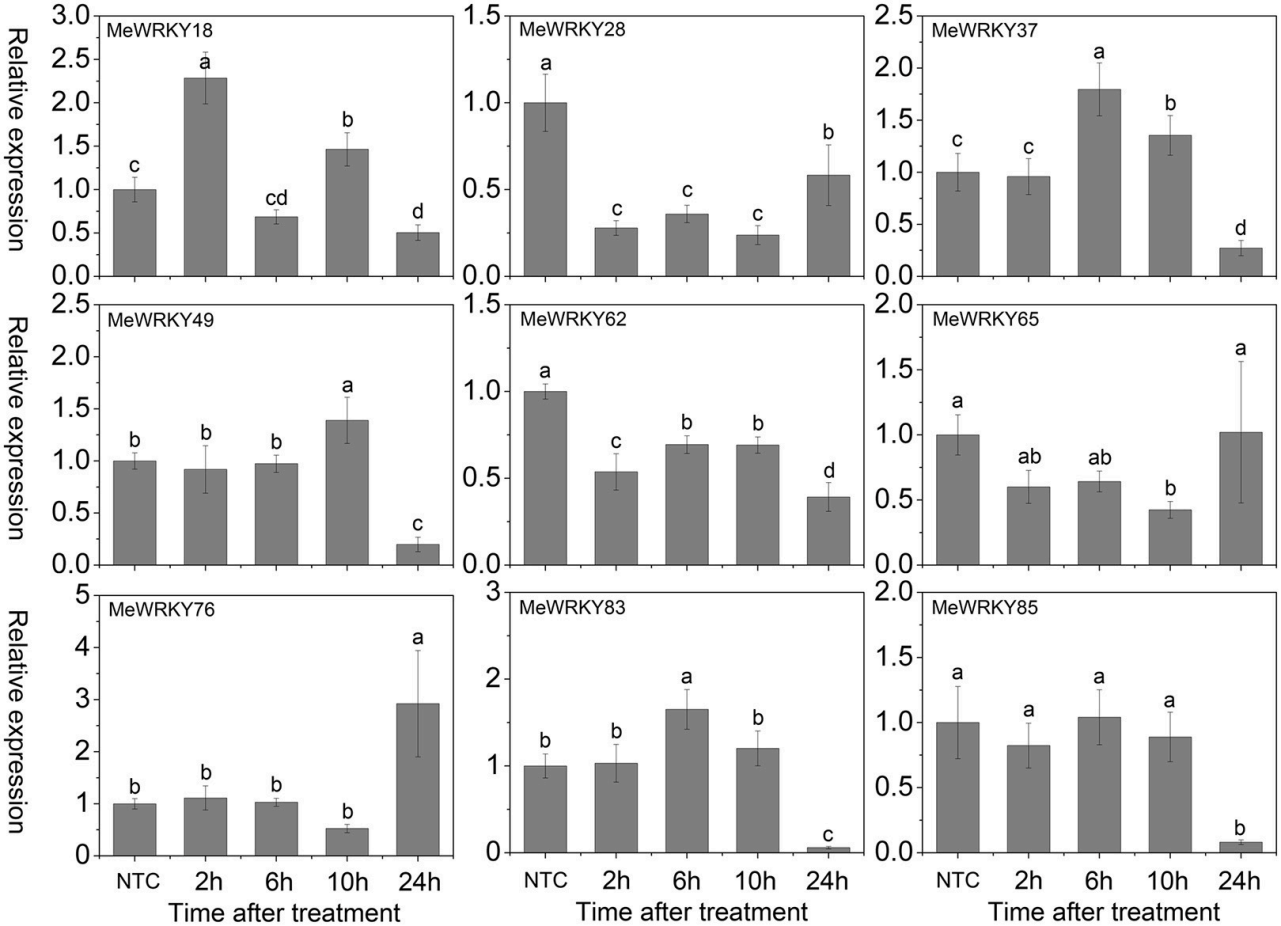
**Expression profiles of ***MeWRKY*** genes in leaves under H_**2**_O_2_ treatment**. The relative expression levels of *MeWRKY* genes in each treated time point were compared with that in each time point at normal conditions. NTC (no treatment control) at each time point was normalized as “1.” Data are means ± SE calculated from three biological replicates. Values with the same letter were not significantly different according to Duncan's multiple range tests (*P* < 0.05, *n* = 3).

H_2_O_2_, a well-known toxic molecule, plays a key role in several biotic and abiotic signaling pathways and its accumulation has been found to be induced by environmental and developmental stimuli (Costa et al., 2010). In Arabidopsis, several *WRKYs*, including *AtWRKY6*, -*22*, -*28*, -*30*, -*46* (Scarpeci et al., 2008), and *AtWRKY25* (Jiang and Deyholos, 2009), are rapidly and highly induced after oxidative stress treatment. Among them, *AtWRKY28* (Babitha et al., 2013) and *AtWRKY30* (Scarpeci et al., 2013) were found to positively regulate oxidative stress tolerance, whereas *AtWRKY25* (Jiang and Deyholos, 2009) acts as a negative regulator of oxidative stress response. In other species, some evidence has suggested that *WRKY* genes play a positive role in response to oxidative stress; for example, silencing of *SlDRW1*, a *WRKY* gene from tomato plants (*Solanum lycopersicum*), increased the sensitivity of transgenic plants to H_2_O_2_ with less chlorophyll content in leaf discs (Liu et al., 2014). Overexpression of *ThWRKY4* in Arabidopsis, a *WRKY* gene from tamarisk (*Tamarix hispida*), enhanced tolerance to oxidative stress (Zheng et al., 2013). To determine whether cassava *WRKY* genes play a role in oxidative stress response, the expression of 9 *MeWRKY* genes in response to H_2_O_2_ was examined. Results suggested that *MeWRKY18* and *MeWRKY37* showed significant up-regulation at 2 and 6 h treatments, respectively. *MeWRKY76* was significantly induced at 24 h, while *MeWRKY49*, -*83*, and -*85* were seriously down-regulated at 24 h. *MeWRKY28* and *MeWRKY62* were strongly repressed at all the treated time-points. *MeWRKY65* did not show obvious trends after H_2_O_2_ treatment (Figure 9). These results suggest that cassava *WRKYs* are likely to be involved in oxidative stress response.

**Figure 10:**
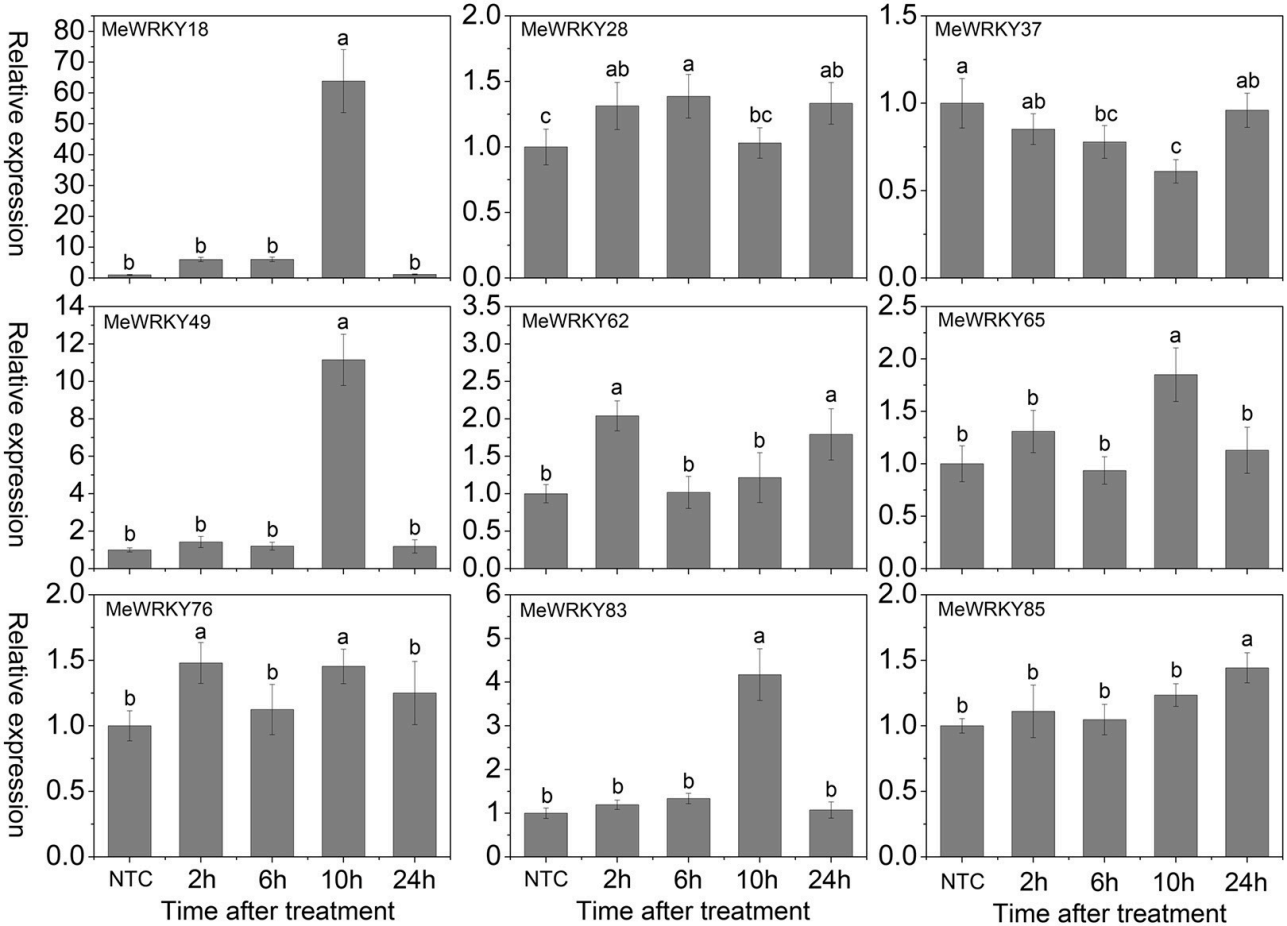
**Expression profiles of ***MeWRKY*** genes in leaves under ABA treatment**. The relative expression levels of *MeWRKY* genes in each treated time point were compared with that in each time point at normal conditions. NTC (no treatment control) at each time point was normalized as “1.” Data are means ± SE calculated from three biological replicates. Values with the same letter were not significantly different according to Duncan's multiple range tests (*P* < 0.05, *n* = 3).

The phytohormone ABA mediates plant responses to abiotic stresses, such as salinity, drought, and cold (Rushton et al., 2010; Mittler and Blumwald, 2015). Evidence has suggested that WRKYs play a crucial role in ABA-mediated signal transduction in plants (Rushton et al., 2010, 2012; Tripathi et al., 2014). In Arabidopsis and rice, several *WRKYs*, including *AtWRKY18* (Chen et al., 2010), *AtWRKY25* (Jiang and Deyholos, 2009), *AtWRKY33* (Jiang and Deyholos, 2009), *AtWRKY40* (Chen et al., 2010), *AtWRKY60* (Chen et al., 2010), *AtWRKY63/ABO3* (Ren et al., 2010), *OsWRKY24*, -*51*, -*71*, and -*77* (Xie et al., 2005), *OsWRKY45-1* and -*45-2* (Tao et al., 2011), *OsWRKY72* (Yu et al., 2010), and *OsWRKY76* (Yokotani et al., 2013) have been shown to be induced after ABA treatment. Among them, *AtWRKY18* (Chen et al., 2010), *AtWRKY60* (Chen et al., 2010), *AtWRKY63/ABO3* (Ren et al., 2010), and *OsWRKY45-2* (Tao et al., 2011) take part in the positive regulation of ABA signaling. To investigate the response of *MeWRKYs* in ABA signaling, the expression of 9 *MeWRKYs* in response to ABA treatment was examined. Results suggested that *MeWRKY18*, -*28*, -*49*, -*62*, -*76*, -*83*, and -*85* expression were induced at all the treated time-points, among which *MeWRKY18*, -*49*, and -*83* showed significant up-regulation at 10 h and *MeWRKY28* was significantly induced at 6 h. *MeWRKY37* expression was repressed at all the treated time-points. *MeWRKY65* was significantly up-regulated at 10 h (Figure 10). The expression levels of *MeWRKY18*, -*49*, and -*83* were over four-fold higher at 10 h ABA treatment, indicating their possible function in ABA signaling.

**Figure 11:**
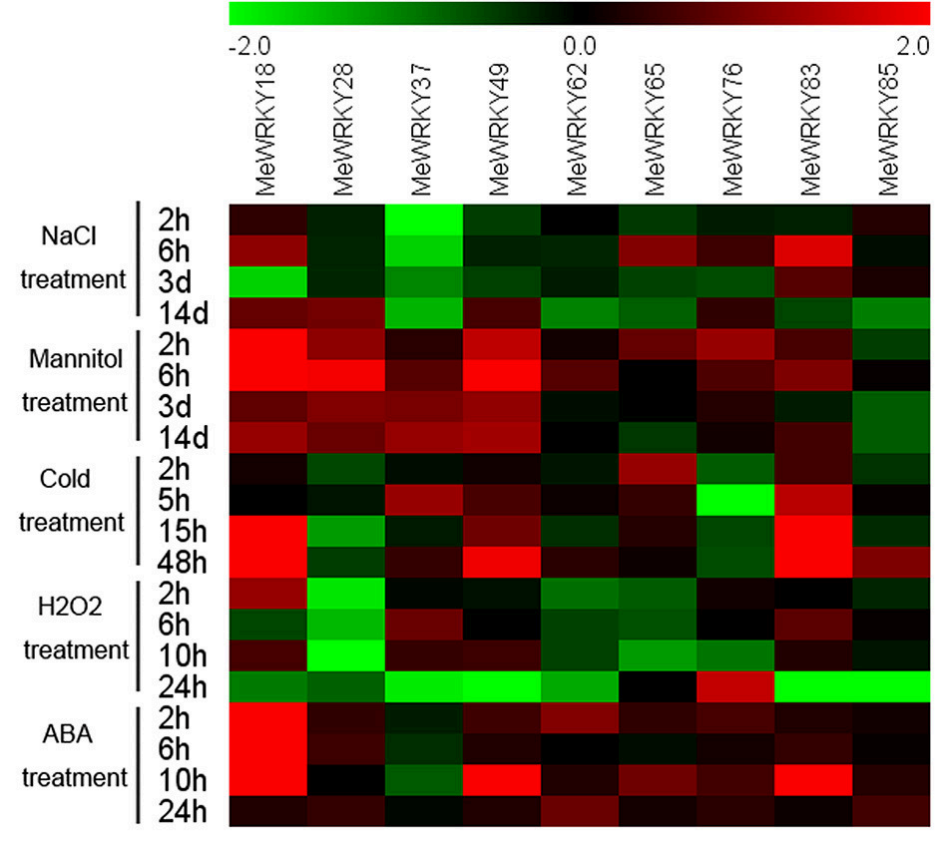
**Expression profiles of ***MeWRKY*** genes in leaves under various stresses and ABA treatments**. Log2 based values from three replicates of qRT-PCR data were used to create the heatmap. The scale represents the relative signal intensity values. Relative expression values for each gene after various treatments are provided in Figures 6–10 and Table S6.

Overall, the patterns in the expression of *MeWRKYs* under various conditions suggest that different *MeWRKY* genes may be involve in different signaling and stress responses, and that a single *MeWRKY* gene also participates in multiple signaling and stress processes. Moreover, most of the cassava *WRKY* genes can be quickly and significantly induced by multiple stressors, ABA, and H_2_O_2_ treatments, indicating that *WRKY* genes may function on multiple transduction pathways in cassava (Figure 11; Table S6).

## Conclusions

In this study, 85 *WRKY* genes from the cassava genome were identified and their basic classification and evolutionary characteristics were established. This information may provide abundant resources for functional characterization of *WRKY* genes. The differential expression patterns of *MeWRKYs* in tissues of the wild subspecies and cultivated varieties revealed that they play different roles in cassava development, and a large number of them exhibited tissue-specific expression, thus assisting in understanding the molecular basis for genetic improvement of cassava. In addition, transcriptomic analysis of different cassava accessions associated with drought stress indicated that the majority of *MeWRKYs* in the root of W14 subspecies were activated in response to drought, which may contribute to its strong tolerance to drought. Furthermore, analysis of the expression of *MeWRKY* genes after various treatments suggested that they have a comprehensive response to osmotic, salt, ABA, H_2_O_2_, and cold, implying that cassava WRKYs may represent convergence points of different signaling pathways. These data may facilitate further investigation of WRKY-mediated signaling transduction pathways. Taken together, this work would provide a solid foundation for future functional investigation of the WRKY family in cassava.

### Availability of supporting data

The cassava WRKY genes identified in this study was submitted to GenBank and the accession number was shown in Table S3. The transcriptomic data was submitted to NCBI and the accession number was shown in Table S7.

## Author contributions

WH, KL, HS, and ZX conceived the study. YW, ZX, WT, ZD, YY, WW performed the experiments and carried out the analysis. WH, YW, and HS designed the experiments and wrote the manuscript. All authors read and approved the final manuscript.

### Conflict of interest statement

The authors declare that the research was conducted in the absence of any commercial or financial relationships that could be construed as a potential conflict of interest.

## Supplementary material

Figure S1 **Sequence logos for conserved motifs identified in MeWRKYs by MEME analysis**.

Table S1 **Primers used in qRT-PCR analysis**.

Table S2 **Characteristics of MeWRKY family in cassava**.

Table S3 **The accession numbers of WRKYs in cassava and Arabidopsis**.

Table S4 **The expression data of the cassava *WRKY* genes in different tissues**.

Table S5 **The expression data (log2-based values) of the cassava *WRKY* genes after drought treatment**.

Table S6 **The expression data (log2-based values) of the cassava *WRKY* genes after various stresses, ABA and H_2_O_2_ treatments**.

Table S7 **The accession number of transcriptomic data in NCBI**.
