# Supplementary figures and images for: Genome-Wide Identification and Expression Analysis of the WRKY Gene Family in Cassava
Source: Front Plant Sci. 2016 Feb 5;7:25. doi: 10.3389/fpls.2016.00025 (PMC4742560; doi:10.3389/fpls.2016.00025)

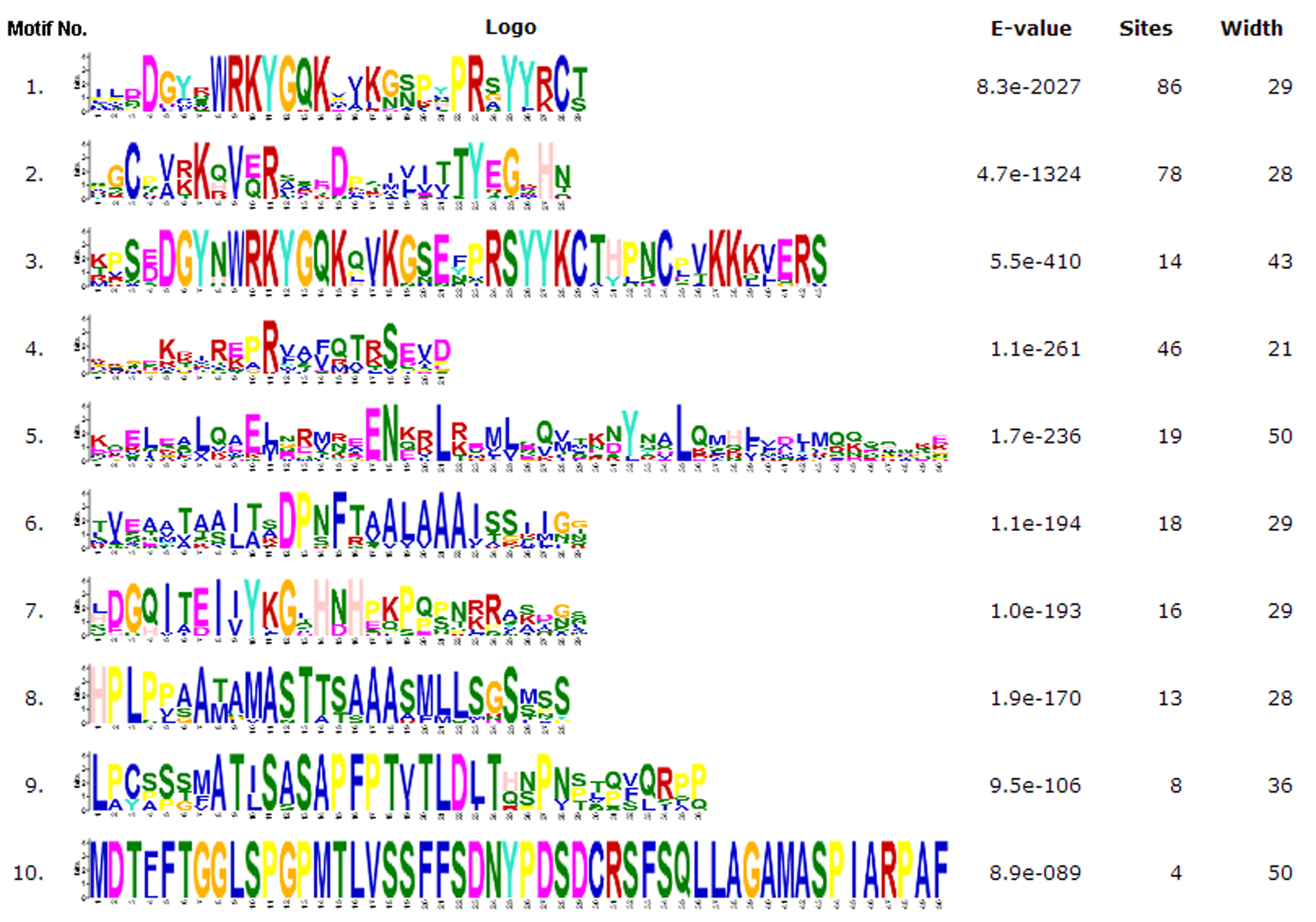

Supplement: Figure S1 — Sequence logos for conserved motifs identified in MeWRKYs by MEME analysis. [file Image1.TIF]
